# Global research trends on maternal exposure to methylmercury and offspring health outcomes

**DOI:** 10.3389/fphar.2022.973118

**Published:** 2022-09-06

**Authors:** Priscila Cunha Nascimento, Maria Karolina Martins Ferreira, Leonardo Oliveira Bittencourt, Paulo Antônio Martins-Júnior, Rafael Rodrigues Lima

**Affiliations:** ^1^ Laboratory of Functional and Structural Biology, Institute of Biological Sciences, Federal University of Para (UFPA), Belém, PA, Brazil; ^2^ Department of Child and Adolescent Oral Health, Federal University of Minas Gerais (UFMG), Belo Horizonte, MG, Brazil

**Keywords:** methylmercury, toxicology, pregnancy, neonatal outcomes, bibliometrics

## Abstract

This study aimed to analyze the landscape of maternal methylmercury exposure and its offspring consequences based on knowledge mapping of the 100 most-cited papers about this theme. A search was performed using the Web of Science, without any restriction of language or publication year. Data bibliometrics, such as the number of citations, citation density, corresponding author’s country, year of publication, study design, and keywords, were extracted from each paper and analyzed. VOSviewer software was used to create graphical bibliometric maps. Of a total of 1,776 studies on this theme, the 100 most-cited papers rendered the number of citations ranged from 110 to 1,356 citations. The non-systematic reviews and cohort studies from Anglo-Saxon countries published in the first decade of the 2000s were the most frequent. Clarkson, Grandjean, and Myers were the authors with higher citation density. A total of 520 keywords represented the evolution of the theme, from classic episodes of MeHg intoxication, as well as main the health changes until the different forms of exposure and, in recent years, biomonitoring studies were highlighted. Our findings provide the global research trends highlighting the network of most influential authors and a better understanding of the evolution and future scenarios of this theme.

## 1 Introduction

Mercury is a metal found in the environment in inorganic forms, elemental mercury and mercury salts such as mercury chloride, and organic forms, such as methylmercury and ethylmercury (Clarkson, 2002; Bernhoft, 2012). Its presence occurs naturally, due to volcanic activities and oceanic resurgences, but it is a major public health problem, mainly due to anthropogenic actions, such as illegal gold mining and inadequate dumping of industrial waste. Among the chemical species of this metal, organic components, especially methylmercury, are highlighted due to their more severe harmful effects (Clarkson, 1997; Bernhoft, 2012; Crespo-Lopez et al., 2021).

As previously reviewed by Grandjean et al. (2010), the first reports of methylmercury poisoning predate the 19th century, occurring in the year 1865, when the first associations of neurological damage to methylmercury exposure were reported (Edwards, 1965). From the 1950s, the methylmercury gained greater repercussion after the case of contamination of Minamata Bay in Japan, during which, through the inadequate dumping of industrial waste in the bay, several cases of chronic intoxication due to the consumption of contaminated fish were evidenced. Since then, clinical manifestations, mainly neurological disorders, resulting from methylmercury intoxication have been named Minamata Disease, which affects adult individuals and also causes damage during pregnancy and child development (Harada, 1995).

The first reported cases of methylmercury poisoning by children and pregnant women were described in 1952 by Engleson and Herner (Engleson and Herner, 1952), and described the case of a family that used contaminated flour in the feeding of one child and during the gestation of another. Severe neurological damage was observed, reported at the time as intellectual disability and motor damage (Grandjean et al., 2010). In parallel, several investigations were carried out in Minamata that also pointed in the same direction of neurological damage generated by exposure to methylmercury during critical phases of nervous system development (Kitamura et al., 1957; Takeuchi, 1964; Harada, 1966). In this sense, the first evidence in experimental studies involving questions about the damage of prenatal exposure was evidenced by Spyker et al. (1972) who reinforced previously reported human cases. Over the years that followed, many other works were published, generating numerous studies about the effects of exposure to methylmercury during the gestational period (Davidson et al., 2004; Solan and Lindow, 2014; Murcia et al., 2016).

Considering the construction of knowledge over the years about mercury toxicology, a bibliometric analysis would allow the mapping of this knowledge generation, as well as the identification of the main evidence, research groups, and/or even the chronology of how this knowledge has been constructed to this day. Thus, this study aimed to identify the top 100 most-cited papers published about maternal exposure to methylmercury and offspring health outcomes.

## 2 Materials and methods

A bibliometric study was performed on 25 May 2021, to search and retrieve the 100 most-cited papers on maternal mercurial exposure and its consequences following previously published methods (Ahmad et al., 2020; Baldiotti et al., 2021).

### 2.1 Search strategy and selection criteria

Based on the research strategy TS = (methylmercury OR “methylmercury compounds”) AND TS = (pregnancy OR “maternal exposure” OR “placental transfer” OR prenatal OR “early exposure”), a search was performed on the electronic database Clarivate Web of Science (WoS) (http://www.webofknowledge.com) choosing the section “All Databases”, without restrictions on publication period and language. First, the records associated with the theme were recovered, and then the list was organized by the number of citations in descending order. Subsequently, they were individually revised for selection. Editorials and conference papers, as well as studies that did not correspond to the specific theme, were excluded. The paper selection stopped at the hundredth most-cited paper, and full-texts and all data were obtained.

The search and extraction of data from the selected studies were carried out by two independent researchers (PCN and LOB), and any discordance was resolved through discussion and consensus with a third researcher (RRL).

### 2.2 Data extraction

The data collected were the paper positions, number of citations, mean number of citations per year (citation density), paper titles, authors (number and names), country, continent, publication year, scientific journal titles, journal impact factor (JIF), journal theme, and study design. The position of papers on the list was based on the highest number of citations in the WoS “All Databases”. The WoS Core Collection (WoS-CC), Scopus, and Google Scholar were consulted to compare the number of citations and citation density. Countries and continents were determined based on the author’s affiliation for correspondence, and the representation of the results was by an adapted map created on mapchart.net (https://mapchart.net/). Study designs were classified into: reviews (systematic or non-systematic), observational (cross-sectional, case-control, cohort or ecological), interventional (clinical studies), and laboratory (*in vitro*, *in vivo*, *in situ*, or *ex vivo*) studies.

### 2.3 Data analysis

#### 2.3.1 Bibliometric networks

The VOSviewer software (version 1.6.16) was used to create the bibliometric net-works (Van Eck and Waltman, 2020). For the co-authorship map, the authors’ names were introduced into the soft-ware as the unit of analysis, and they were linked to each other based on the number of jointly authored papers. These results were present by network and density visualizations. For the co-occurrence map, all keywords were introduced into the software as the unit of analysis. Based on this, the results were showed in overlay visualization.

In the networks, the software assigns the nodes to clusters, which is a set of closely related nodes characterized by colors, circles, lines, and the number of clusters. Each cluster is represented by a color. More important terms are larger circles, and strongly related terms are closer to each other. The lines between terms indicate relations and, when thicker, represent a stronger link between two terms. The number of clusters is determined by a resolution parameter—the higher the value of this parameter, the larger the number of clusters (Van Eck and Waltman, 2010).

#### 2.3.2 Statistical analysis

The descriptive data and associations between the number of citations, JIF, and study design were analyzed. The descriptive analysis was performed using electronic spread-sheets (Microsoft Excel^®^, Windows 10 version). The ratio between the number of citations and the number of articles was estimated for each country (citation ratio, CR). Bivariate analysis was performed using the Statistical Package for the Social Sciences (SPSS; version 24.0; IBM, Chicago, IL, EUA). For this, the Kolmogorov-Smirnov test was used to assess the normality of data distribution. After that, the number of citations (at different databases) and JIF were analyzed by Pearson’s correlation. The study design was categorized by evidence level (literature review, experimental animal study, case report or case series, cross-section, case-control, cohort, and systematic review) and Spearman’s rank correlation coefficient test. The significance level was set at 5%.

## 3 Results

### 3.1 Number of citations and citation density

The search strategy resulted in 1,776 papers and was organized by the list of descending number of citations. A total of 127 papers were revised for eligibility, of which 27 were excluded (Supplementary Table S1). Altogether, the 100 most-cited papers were cited 29,208 times in Web of Science (WoS) “All Databases”, ranging from 110 to 1,356 citations each, and the first seven of them were cited more than a thousand times, with a variation of 24.47–96.86 citations/year (mean: 68.35 citations/year).

When compared to other databases, the number of paper citations was higher in Google Scholar (46,645 citations), followed by Scopus (30,450 citations), as well as means of citation density (means: 30.59 and 20.13 citations/year, respectively) (Supplementary Table S2). Strong positive correlations statistically significant were observed between the number of citations in WoS and Scopus (r = 0.998, *p* < 0.0001), and WoS and Google Scholar (r = 0.988, *p* < 0.0001).

### 3.2 Characteristics of the 100 most-cited papers

The most-cited paper was a non-systematic review study, entitled “The toxicology of mercury and its chemical compounds”, authored by Clarkson and Magos, and published in Critical Reviews in Toxicology in 2006. This study was cited 1,356 times in WoS “All Databases” (mean: 96.86 citations/year), receiving 1,317 citations in the WoS-CC, 1,409 in Scopus, and 2,027 in Google Scholar.

Curiously, most of the most-cited papers were also from the first decade of the 21st century (50 papers; 14,581 citations). Just as the most-cited paper, the study design most prevalent was non-systematic review, then the cohort and cross-sectional studies. There was no significant correlation between a higher level of evidence and the number of citations (r = −0.046, *p* = 0.674). Table 1 summarizes the main characteristics of the top 100 most-cited papers.

**TABLE 1:** Frequencies of characteristics of the 100 most-cited papers in maternal exposure to methylmercury and offspring health outcomes.

Characteristics	Number of papers	Number of citations^a^
Period of time of publication		
1972–1979	11	3,043
1980–1989	6	1,078
1990–1999	18	6,547
2000–2009	50	14,581
2010–2017	15	3,959
Number of authors		
1–2	19	9,856
3–4	27	6,436
5–6	20	4,348
>6	34	8,568
Study design		
Laboratorial studies (*in vivo*)	8	1,173
Case report	4	767
Case series	1	142
Cross-sectional	12	3,285
Case-control	3	761
Cohort	32	7,367
Non-systematic review	38	15,307
Systematic review		
Without meta-analysis	1	129
With meta-analysis	1	277

a Number of citations in web of science “all databases” section.

**FIGURE 1:**
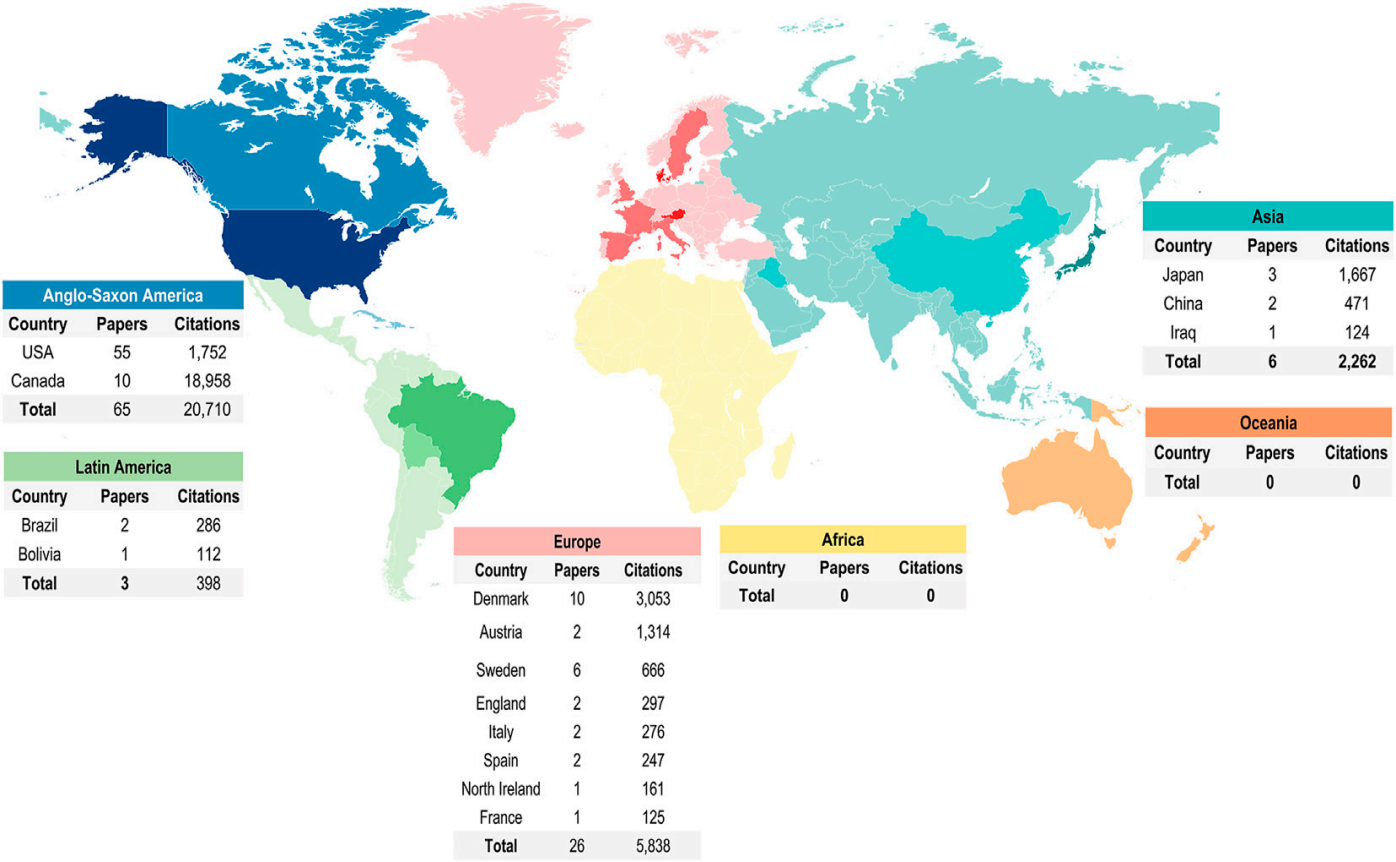
Worldwide distribution of the top 100 most-cited papers in maternal exposure to MeHg and offspring health outcomes.

For institutional location, Anglo-Saxon America and Europe were the continents with most papers. The United States and Canada widely led the ranking of countries, and both represented more than half of the 100 most-cited papers. Despite this, Japan (CR = 555.67) was the first country with significant numbers of citations per paper, followed by the United States (CR = 344.69) and Denmark (CR = 324.70). On the other hand, a lower distribution in the other countries was observed, as shown in Figure 1.

In general, Environmental Health Perspectives (15%), Neurotoxicology (8%), Environmental Research (7%), Neurotoxicology and Teratology (5%), and Science of the Total Environment (5%) were the journals with the most papers on the top 100 list. The Neurotoxicology was pioneer in this theme. On the other hand, Environmental Health Perspectives and Environmental Research were the only journals that maintained the publication of most-cited papers in the last decade. Another 43 journals published 1–4 of the top 100 papers about the field of neurotoxicology and environmental/experimental toxicology. It is interesting to highlight that some of the most-cited papers about maternal exposure to MeHg and offspring health outcomes were published in some journals of high impact factor in health sciences, such as The Lancet (2 papers), Science (2 papers), Journal of The American Medical Association (JAMA) (2 papers), and The New England Journal of Medicine (1 paper). A moderately positive statistically significant correlation was observed between the number of citations and JIF (r = 0.550, *p* < 0.0001).

### 3.3 Networks of researchers connected by co-authorship

**FIGURE 2:**
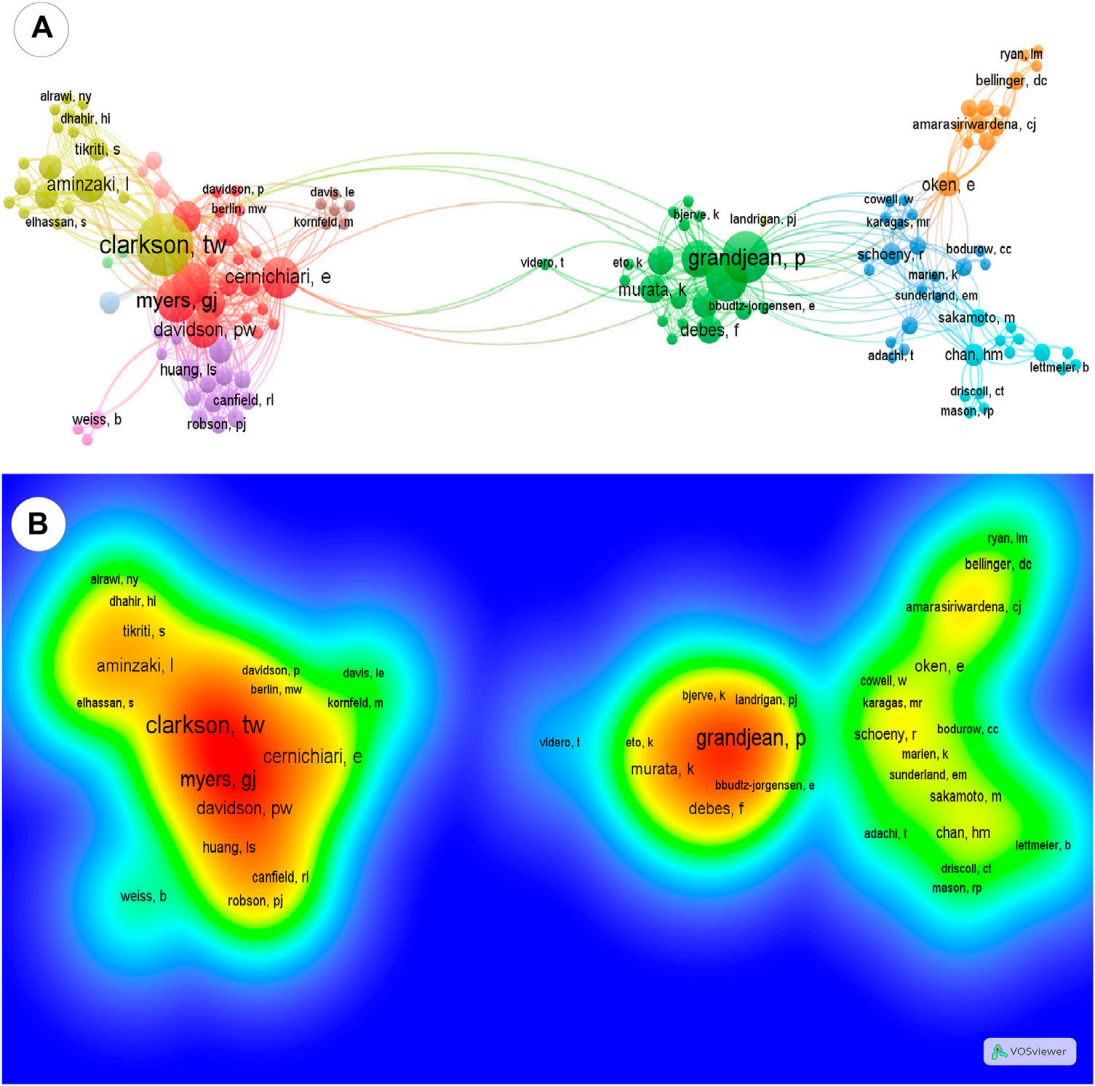
Network of co-authorship and density citation view of authors. **(A)** Network visualization of the top 100 most-cited papers about maternal exposure to MeHg and offspring health outcomes: same colors represent the same cluster, and lines between clusters are co-citation links between the authors. The closer the authors are located to each other, the stronger their relatedness. **(B)** Density visualization: colors indicate the citation density of authors, ranging from blue (lowest density) to red (highest density).

Papers published by more than six (34%) and three to four (27%) authors were the most frequent ones (Table 1). A total of 382 authors were identified in the top 100 papers. The authors with the greatest total link were selected, and the VOSviewer map detailed the co-authorship relationships among authors (Figure 2A) and the citation density (Figure 2B) through clusters. The main clusters of high citation density contained prominent authors, such as Clarkson, Myers, and Cernichiari gathering other contributors. Another large cluster was constituted by Grandjean gathering three different clusters of researchers.

### 3.4 Network of keywords connected by co-occurrence

**FIGURE 3:**
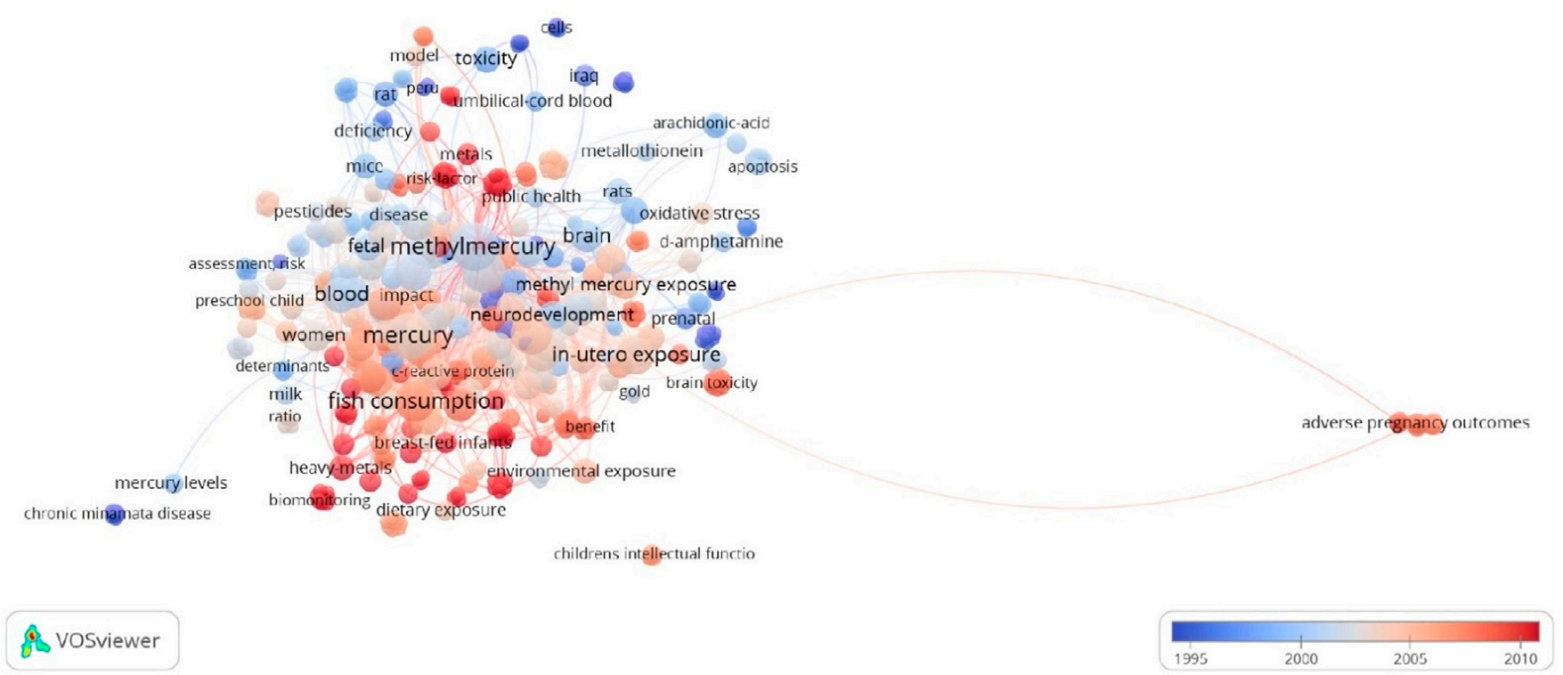
Overlay visualization of keywords connected by co-occurrence. Network of the all-keywords distribution along the years of publications (color scale) of the top 100 most-cited papers about maternal exposure to MeHg and offspring health outcome.

In all, 520 keywords were identified. The most commonly used keyword was mercury (*n* = 39), followed by methylmercury (*n* = 34), prenatal exposure (*n* = 22), fish consumption (*n* = 21) and *in utero* exposure (*n* = 17). The distribution of the keywords along the years of publications is shown in Figure 3.

## 4 Discussion

This bibliometric study presents evidence that literature review studies from Anglo-Saxon countries and published in the first decade of the 2000s were the most-cited papers in maternal exposure to methylmercury and offspring health outcomes. This analysis was based on the 100 most-cited papers and highlighted the mapping of global research trends of this theme, both about the network of authors and the evolution of the research focus based on the keywords.

The number of citations is used to measure the impact of papers, journals, and researchers and is often incorporated into academic advancement decisions (Kulkarni et al., 2009). Thus, it is necessary to use a proper database since each one uses particular methods to record and count citations (Kulkarni et al., 2009; de Granda-Orive et al., 2011). The Web of Science remains one of the best tools available for analyzing citations due to the possibility of retrieving publications since 1945, in addition, it covers high-quality peer-reviewed academic journals published worldwide (Bakkalbasi et al., 2006; Falagas et al., 2008). Based on this, our bibliometric study used this database, and there was a positive statistical correlation in the number of citations between WoS, Scopus, and Google Scholar, despite previous reports about numerical differences between these databases in the other search fields (Bakkalbasi et al., 2006; Kulkarni et al., 2009). Our finding can be characterized as particular to the toxicology area, especially about the theme investigated.

To the best of our knowledge, the publication metrics on exposure to mercury themes have not been previously addressed in the toxicology field. Other papers have investigated the bibliographic metrics of a specific journal (Zyoud et al., 2014a), region (Miró et al., 2009; Zyoud et al, 2014b), or metal (Hu et al., 2010). This present bibliometric review is the first citation analysis of the theme in human environmental toxicology of methylmercury, mainly in the early stages of life.

A basic theory about citation analysis is that the more cited a study, the greater its influence on the theme (Garfield and Merton, 1979; Martínez et al., 2014). Usually, a paper is a citation classic when it is cited at least 400 times, and can highlight the main intellectual markers of the research field, either from a historical perspective or by identifying relevant journals, countries, or researchers (Moed, 2009; Martínez et al., 2014). Here, fourteen papers received 400 or more citations and seven studies received more than 1,000 citations. The achieved impressive metrics demonstrate the considerable interest in investigating maternal mercurial exposure and its consequences.

Concerning the historical perspective, the citation classics identified in our review presents different temporal milestones. Analyzing these papers, their impressive metrics may be due to their older years of publication and reporting historical markers of environmental exposure to methylmercury; thus, a high citation number would be expected. In our review, the oldest study with humans described the epidemic of methylmercury poisoning in farmers and their families in Iraq (Bakir et al., 1973); the second and the third most-cited papers reviewed the damages resulting from Minamata disease in Japan, which occurred in humans who ingested fish and shellfish contaminated with MeHg discarded in wastewater from a chemical plant (Harada, 1995). Curiously, it was evidenced also among the citation classics the scene current of global concern about the theme, from studies as Driscoll’s (Driscoll et al., 2013) and Clarkson’s (Clarkson et al., 2003) papers.

Another bibliographic metric commonly analyzed is the identification of relevant journals by impact factor (Garfield and Merton, 1979; Martínez et al., 2014). Comparing toxicology to areas where new findings are made almost daily, such as fields of molecular biology and genetics, human toxicology is a slower advancing science (Ioannidis, 2006; Zyoud et al., 2014a). Thus, there are wide disparities between the number of citations of journals in health sciences in comparison to a journal of a specific field (Jones, 2003; Zyoud et al., 2014a). This may be one reason why we also observed a moderate correlation between a greater number of citations of journals with a greater JIF, such as The Lancet, Science, JAMA, and The New England Journal of Medicine. However, in the top 100 most-cited papers in our review, journals with the scope of toxicology prevailed, such as Environmental Health Perspectives, Neurotoxicology, Environmental Research, Neurotoxicology and Teratology and Science of the Total Environment.

The most-cited paper was published by (Clarkson and Magos, 2006), receiving 1,356 citations. This document is a review paper, as were most papers in the top 100 most-cited list. Normally, this occurs because the journals that publish more reviews tend to present higher citation rates than original papers, as previously reported by other authors (Ioannidis, 2006; Allareddy et al., 2012; Royle et al., 2013; Zyoud et al., 2014a). Besides, cohorts were the second most prevalent study design, which may be associated with the need for the time-response factor in maternal methylmercury exposures to analyze the fetal outcomes.

The most-cited paper is from the United States, which was also the country with the largest number of papers in our bibliometric review, being responsible for 65% of corresponding authors’ addresses all documents. Similarly, it was also the most frequent home country of other bibliometric studies in toxicology (Jones, 2003; Hu et al., 2010; Zyoud et al., 2014a), despite the steady increase of publication by other countries, mainly of the European and Asian continents (Miró et al., 2009; Zyoud et al., 2014b). This United States leadership can be explained by their hosting the world’s leading main research centers, where more funding is invested (Ioannidis, 2006; Zyoud et al., 2014a). Besides that, Japan was the first in the number of citations per paper, thus presenting the highest citation ratio (CR), which may be directly related to the frequently investigated Minamata disease (James et al., 2020).

On the other hand, these more frequent studies from Anglo-Saxon countries may represent a weakness in the thematic here investigated. Maternal exposure to methylmercury and possible repercussions on the fetus is a topic of global relevance since it is present in several governmental environmental reports (WHO, 2014a; WHO, 2014b; UNEP, 2018; WHO, 2021). Thus, this high concentration of the number of citations can represent publication barriers and gaps in networks of researchers with other relevant regions. According to the data analyzed, Grandjean is a Danish scientist who has established contributions with the United States authors, such as Clarkson, Myers, and Cernichiari. Thus, these networks of successful co-authorships can be associated with the larger citation density of these authors (see Figure 3). Based on this, more contributions between different research groups should be encouraged.

In this bibliometric analysis, we highlight the network of keywords connected by co-occurrence in the top 100 most-cited papers, based on the frequency of these terms along the years of publications (Figure 3). Historically, terms that refer to classic episodes of methylmercury intoxication are more frequent (“chronic Minamata disease,” “Iraq”), as well as main initial study models (“cell,” “rats,” “mice”). Over the years, keywords associated with the damage mechanisms of this mercurial exposure start to emerge, such as “oxidative stress,” “apoptosis,” “metallothionein,” and “c-reactive protein”. Besides, the central nervous system, mainly during development, becomes the object of study most frequently indexed, through keywords “brain”, “neurodevelopment,” and “brain toxicity”. Another evolution observed is the terms related to the forms of exposure: initially, “pesticides” appear among the indexations, passing through “gold,” “environmental exposure,” and more recently “dietary exposure” and “fish consumption”. Currently, the use of keywords that reflect debates necessary for damage management is noticeable, such as “biomonitoring,” “public health,” and “risk factor”. This mapping allowed us to analyze the global research trends of this theme being able to help editors and researchers in comments about current and new research lines.

According to the main findings of the studies, mercury is capable to change various neurological functions, such as language, attention, memory, visuospatial and motor functions (Grandjean et al., 1997; Debes et al., 2006). Another study points out that high levels of mercury are related to cognitive impairment (Oken et al., 2005). In addition, studies show that the main source of mercury exposure is through contaminated foods such as fish and shellfish. Evidence shows that consumption of these foods with significant levels of methylmercury during the prenatal period was associated with a deficit in neurological development (Steuerwald et al., 2000). It is important to emphasize that methylmercury can cross the placental barrier and compromise the fetal nervous system at a crucial period of maturation (Harada, 1995; Rice and Barone, 2000).

High levels of mercury can alter brain size, promote damage in different brain regions such as cortex, hippocampus, change myelin content, and even cell death (Burbacher et al., 1990). Exposure to methylmercury even at levels considered low can trigger depressive symptoms and modulate the expression of important neurotrophins such as BDNF in the hippocampus (Onishchenko et al., 2008). Furthermore, studies show that in the CNS, methylmercury easily accumulates in astrocytes, altering the uptake of glutamate by these cells (Aschner et al., 2000).

The present results are an advance for toxicological reports, but they must be interpreted with some limitations. The assessment of self, direct, and co-author citations was not performed. In parallel, it stands out that the corresponding author affiliation was determined the home country of the papers; however, we know that collaboration between research groups can include authors from different institutions and countries. Besides, there may be some missing relevant studies published before 1945 since WoS is not able to include papers published before this period.

Overall, the present study is the first to report the bibliometric characteristics and research trends of the papers about methylmercury toxicology, specifically the pre and postnatal exposure and its consequences on neonates. Thus, this review synthesized useful metrics for journal editors and researchers to better understand the scientific advancement and trends of papers in the international scientific scene.

## Data Availability

The original contributions presented in the study are included in the article/Supplementary Material, further inquiries can be directed to the corresponding author.
