# Supplementary material for: Global research trends on maternal exposure to methylmercury and offspring health outcomes
Source: Front Pharmacol. 2022 Sep 6;13:973118. doi: 10.3389/fphar.2022.973118 (PMC9485893; doi:10.3389/fphar.2022.973118)
Supplement: Supplementary file 1 [file Table1.DOCX]

*Supplementary Material*

Global research trends on maternal exposure to methylmercury and offspring health outcomes

Priscila Cunha Nascimento^1^, Maria Karolina Martins Ferreira^1^, Leonardo Oliveira Bittencourt^1^, Paulo Antônio Martins-Júnior^2^, Rafael Rodrigues Lima^1^*

^1^Laboratory of Functional and Structural Biology, Institute of Biological Sciences, Federal University of Para, Belém, PA, Brazil

^2^Department of Child and Adolescent Oral Health, Federal University of Minas Gerais (UFMG), Belo Horizonte, MG, Brazil

*Correspondence: Rafael Rodrigues Lima, PhD rafalima@ufpa.br; Laboratory of Functional and Structural Biology, Institute of Biological Sciences, Federal University of Pará, 01 Augusto Correa Street, Campus Guamá, Belém, Pará, 66075-900, Brazil

This Supplementary Material contains 1 table.

**Supplementary Table 1.** Excluded papers with reason

| Citation number in WoS ‘All database’ | Paper | Reason |
| --- | --- | --- |
| 522 | Goyer RA. Toxic and essential metal interactions. Annu Rev Nutr. 1997;17:37-50. | Debate interactions between toxics harmful to cognitive development |
| 421 | Vahter M, Akesson A, Lidén C, Ceccatelli S, Berglund M. Gender differences in the disposition and toxicity of metals. Environ Res. 2007;104(1):85-95. | Exposure in different genres without debating the repercussions on offspring |
| 292 | Koletzko B, Cetin I, Brenna JT, et al. Dietary fat intakes for pregnant and lactating women. Br J Nutr. 2007;98(5):873-877. | Recommendations on dietary fat intake during pregnancy and lactation |
| 289 | Ralston NV, Raymond LJ. Dietary selenium's protective effects against methylmercury toxicity. Toxicology. 2010;278(1):112-123. | Debates the status of selenium (Se) in the diet is inversely related to vulnerability to methylmercury (MeHg) toxicity. |
| 261 | Fitzgerald WF, Clarkson TW. Mercúrio e monometilmercúrio: preocupações presentes e futuras. Perspectiva de saúde da Environ . 1991; 96: 159-166. | Debate about the Hg cycle, harm to humans. No direct correlation between maternal exposure and offspring health effects |
| 249 | Grandjean P, Weihe P, Burse VW, et al. Neurobehavioral deficits associated with PCB in 7-year-old children prenatally exposed to seafood neurotoxicants. Neurotoxicol Teratol. 2001;23(4):305-317. | Prenatal exposure to polychlorinated biphenyls (PCBs) was examined by analyzing umbilical cord tissue from 435 infants from a Faroese birth cohort. |
| 247 | Chang LW. Neurotoxic effects of mercury--a review. Environ Res. 1977;14(3):329-373. | Does not debate damage during neurodevelopment |
| 239 | Liu Y, Chen M, Cao T, et al. A cyanine-modified nanosystem for in vivo upconversion luminescence bioimaging of methylmercury. J Am Chem Soc. 2013;135(26):9869-9876. | System validation for MeHg biomonitoring |
| 211 | Grandjean P, White RF, Nielsen A, Cleary D, de Oliveira Santos EC. Methylmercury neurotoxicity in Amazonian children downstream from gold mining. Environ Health Perspect. 1999;107(7):587-591. | Does not correlate with prenatal exposure; Only mother-child ratio of 7-12 years old |
| 187 | Govarts E, Nieuwenhuijsen M, Schoeters G, et al. Birth weight and prenatal exposure to polychlorinated biphenyls (PCBs) and dichlorodiphenyldichloroethylene (DDE): a meta-analysis within 12 European Birth Cohorts. Environmental health perspectives, 2012;120(2), 162-170. | Prenatal exposure to polychlorinated biphenyls (PCBs) and dichlorodiphenyldichloroethylene (DDE) |
| 187 | Oken E, Kleinman KP, Berland WE, Simon SR, Rich-Edwards JW, Gillman MW. Decline in fish consumption among pregnant women after a national mercury advisory. Obstet Gynecol. 2003;102(2):346-351. | Pattern of fish intake by pregnant women after government reports; No discussion of maternal-fetal exposure relationship |
| 176 | Thompson WW, Price C, Goodson B, et al. Early thimerosal exposure and neuropsychological outcomes at 7 to 10 years. New England Journal of Medicine. 2007;357(13), 1281-1292. | Mercury from vaccines containing thimerosal and neuropsychological deficit at age 7 to 10 years |
| 175 | Goldman LR, Shannon MW; American Academy of Pediatrics: Committee on Environmental Health. Technical report: mercury in the environment: implications for pediatricians. Pediatrics. 2001;108(1):197-205. | General review on Hg (exposure sources, types of Hg, toxicokinetics, etc.); No discussion of maternal-fetal exposure relationship |
| 172 | Cohen JT, Bellinger DC, Connor WE, et al. A quantitative risk-benefit analysis of changes in population fish consumption. Am J Prev Med. 2005;29(4):325-334. | Pattern of fish intake by pregnant women versus general population |
| 169 | Kaneko JJ, Ralston NV. Selenium and mercury in pelagic fish in the central north pacific near Hawaii. Biol Trace Elem Res. 2007;119(3):242-254. | Debate protective effects of selenium against mercury toxicity |
| 151 | Burns G, Brooks K, Wildung M, Navakanitworakul R, Christenson LK, Spencer TE. Extracellular vesicles in luminal fluid of the ovine uterus. PLoS One. 2014;9(3):e90913. Published 2014 Mar 10. | Compares concentrations and total stocks of Hg and methyl Hg in soil from different landscapes in two watersheds |
| 150 | Grandjean P, Weihe P, Needham LL, et al. Relation of a seafood diet to mercury, selenium, arsenic, and polychlorinated biphenyl and other organochlorine concentrations in human milk. Environ Res. 1995;71(1):29-38. | Investigate Hg concentration in breast milk. No evaluations with outcome in offspring |
| 149 | Aschengrau A, Zierler S, Cohen A. Quality of community drinking water and the occurrence of spontaneous abortion. Arch Environ Health. 1989;44(5):283-290. | Correlates drinking water quality with spontaneous abortion. Many confounding variables (many other metals and chemical compounds) |
| 141 | Stewart PW, Lonky E, Reihman J, Pagano J, Gump BB, Darvill T. The relationship between prenatal PCB exposure and intelligence (IQ) in 9-year-old children. Environ Health Perspect. 2008;116(10):1416-1422. | Debate prenatal exposure to polychlorinated biphenyls (PCBs) and cognitive deficits in early childhood |
| 141 | Takser L, Mergler D, Baldwin M, de Grosbois S, Smargiassi A, Lafond J. Thyroid hormones in pregnancy in relation to environmental exposure to organochlorine compounds and mercury. Environ Health Perspect. 2005;113(8):1039-1045. | Inorganic Hg (pesticides) |
| 137 | Mozaffarian D. Fish, mercury, selenium and cardiovascular risk: current evidence and unanswered questions. Int J Environ Res Public Health. 2009;6(6):1894-1916. doi:10.3390/ijerph6061894 | Review of health effects of fish consumption in adults |
| 137 | König A, Bouzan C, Cohen JT, et al. A quantitative analysis of fish consumption and coronary heart disease mortality. Am J Prev Med. 2005;29(4):335-346. | Risk benefit on fish intake |
| 133 | Booth S, Zeller D. Mercury, food webs, and marine mammals: implications of diet and climate change for human health. Environ Health Perspect. 2005;113(5):521-526. | Model to predict methylmercury concentrations in fish species based on trophic level variables |
| 132 | Roman HA, Walsh TL, Coull BA, et al. Evaluation of the cardiovascular effects of methylmercury exposures: current evidence supports development of a dose-response function for regulatory benefits analysis. Environ Health Perspect. 2011;119(5):607-614. | Review of the effects of exposure to MeHg on cardiovascular health through consumption of fish and seafood with a focus on adults |
| 128 | Bose-O’Reilly S, Lettmeier B, Gothe RM, et al. Mercury as a serious health hazard for children in gold mining areas. Environmental research. 2008; 107(1), 89-97. | Debate on child labor with hazardous substances such as mercury |
| 114 | Kempson IM, Lombi E. Hair analysis as a biomonitor for toxicology, disease and health status. Chem Soc Rev. 2011;40(7):3915-3940. | Hair debate as a biomarker in toxicology |
| 114 | Myren M, Mose T, Mathiesen L, Knudsen LE. The human placenta--an alternative for studying foetal exposure. Toxicol In Vitro. 2007;21(7):1332-1340. | Placental debate as an object of study for environmental exposure |
