# Supplementary material for: Global research trends on maternal exposure to methylmercury and offspring health outcomes
Source: Front Pharmacol. 2022 Sep 6;13:973118. doi: 10.3389/fphar.2022.973118 (PMC9485893; doi:10.3389/fphar.2022.973118)
Supplement: Supplementary file 2 [file Table2.DOCX]

**Table S2.** All data of 100 most‐cited papers in maternal exposure to methylmercury and offspring health outcomes.

|  |  | Citation number (citation density^a^) | | | |
| --- | --- | --- | --- | --- | --- |
| *Rank* | ***Paper*** | WoS  All databases | WoS  CC | Scopus | Google Scholar |
| 1 | Clarkson TW, Magos L. The toxicology of mercury and its chemical compounds. Crit Rev Toxicol. 2006;36(8):609-662. | 1356 (96.86) | 1317 (94.07) | 1409 (100.64) | 2027 (144.79) |
| 2 | Mozaffarian D, Rimm EB. Fish intake, contaminants, and human health: evaluating the risks and the benefits [published correction appears in JAMA. 2007 Feb 14;297(6):590). JAMA. 2006;296(15):1885-1899. | 1337 (95.50) | 1290 (92.14) | 1423 (101.64) | 2261 (161.50) |
| 3 | Harada M. Minamata disease: methylmercury poisoning in Japan caused by environmental pollution. Crit Rev Toxicol. 1995;25(1):1-24. | 1268 (50.72) | 1222 (48.88) | 1298 (51.92) | 2002 (80.08) |
| 4 | Clarkson TW, Magos L, Myers GJ. The toxicology of mercury--current exposures and clinical manifestations. N Engl J Med. 2003;349(18):1731-1737. | 1231 (72.41) | 1170 (68.82) | 1298 (76.35) | 1821 (107.12) |
| 5 | Grandjean P, Landrigan PJ. Developmental neurotoxicity of industrial chemicals. Lancet. 2006;368(9553):2167-2178. | 1215 (86.79) | 1187 (84.79) | 1283 (91.64) | 1932 (138.00) |
| 6 | Grandjean P, Weihe P, White RF, et al. Cognitive deficit in 7-year-old children with prenatal exposure to methylmercury. Neurotoxicol Teratol. 1997;19(6):417-428. | 1190 (51.74) | 1167 (50.74) | 1271 (55.26) | 1994 (86.70) |
| 7 | Bakir F, Damluji SF, Amin-Zaki L, et al. Methylmercury poisoning in Iraq. Science. 1973;181(4096):230-241. | 1150 (24.47) | 1127 (23.98) | 1107 (23.55) | 1945 (41.38) |
| 8 | Driscoll CT, Mason RP, Chan HM, Jacob DJ, Pirrone N. Mercury as a global pollutant: sources, pathways, and effects. Environ Sci Technol. 2013;47(10):4967-4983. | 979 (139.86) | 956 (136.57) | 1007 (143.86) | 1270 (181.43) |
| 9 | Grandjean P, Landrigan PJ. Neurobehavioural effects of developmental toxicity. Lancet Neurol. 2014;13(3):330-338. | 786 (131.00) | 771 (128.50) | 835 (139.17) | 1256 (209.33) |
| 10 | Clarkson TW. The three modern faces of mercury. Environ Health Perspect. 2002;110 Suppl 1(Suppl 1):11-23. | 708 (39.33) | 682 (37.89) | 756 (42.00) | 1202 (66.78) |
| 11 | Clarkson TW. The toxicology of mercury. Crit Rev Clin Lab Sci. 1997;34(4):369-403. | 591 (25.70) | 573 (24.91) | 630 (27.39) | 956 (41.57) |
| 12 | Grandjean P, Weihe P, White RF, Debes F. Cognitive performance of children prenatally exposed to "safe" levels of methylmercury. Environ Res. 1998;77(2):165-172. | 547 (24.86) | 535 (24.32) | 568 (25.82) | 763 (34.68) |
| 13 | Davidson PW, Myers GJ, Cox C, et al. Effects of prenatal and postnatal methylmercury exposure from fish consumption on neurodevelopment: outcomes at 66 months of age in the Seychelles Child Development Study. JAMA. 1998;280(8):701-707. | 497 (22.59) | 484 (22.00) | 546 (24.82) | 787 (35.77) |
| 14 | Myers GJ, Davidson PW, Cox C, et al. Prenatal methylmercury exposure from ocean fish consumption in the Seychelles child development study. Lancet. 2003;361(9370):1686-1692. | 411 (24.18) | 400 (23.53) | 452 (26.59) | 689 (40.53) |
| 15 | Karagas MR, Choi AL, Oken E, et al. Evidence on the human health effects of low-level methylmercury exposure. Environ Health Perspect. 2012;120(6):799-806. | 392 (49.00) | 383 (47.88) | 393 (49.13) | 555 (69.38) |
| 16 | Oken E, Wright RO, Kleinman KP, et al. Maternal fish consumption, hair mercury, and infant cognition in a U.S. Cohort. Environ Health Perspect. 2005;113(10):1376-1380. | 369 (24.60) | 361 (24.07) | 395 (26.33) | 582 (38.80) |
| 17 | Zhang L, Wong MH. Environmental mercury contamination in China: sources and impacts. Environ Int. 2007;33(1):108-121. | 348 (26.77) | 304 (23.38) | 322 (24.77) | 498 (38.31) |
| 18 | Counter SA, Buchanan LH. Mercury exposure in children: a review. Toxicol Appl Pharmacol. 2004;198(2):209-230. | 338 (21.13) | 321 (20.06) | 369 (23.06) | 584 (36.50) |
| 19 | Mahaffey KR, Clickner RP, Bodurow CC. Blood organic mercury and dietary mercury intake: National Health and Nutrition Examination Survey, 1999 and 2000. *Environ Health Perspect*. 2004;112(5):562-570. | 329 (20.56) | 324 (20.25) | 351 (21.94) | 539 (33.69) |
| 20 | Clarkson TW. Mercury: major issues in environmental health. Environ Health Perspect. 1993;100:31-38. | 327 (12.11) | 317 (11.74) | 413 (15.30) | 640 (23.70) |
| 21 | Choi BH, Lapham LW, Amin-Zaki L, Saleem T. Abnormal neuronal migration, deranged cerebral cortical organization, and diffuse white matter astrocytosis of human fetal brain: a major effect of methylmercury poisoning in utero. J Neuropathol Exp Neurol. 1978;37(6):719-733. | 306 (7.29) | 304 (7.24) | 304 (7.24) | 428 (10.19) |
| 22 | Amin-Zaki L, Elhassani S, Majeed MA, Clarkson TW, Doherty RA, Greenwood M. Intra-uterine methylmercury poisoning in Iraq. Pediatrics. 1974;54(5):587-595. | 302 (6.57) | 299 (6.50) | 286 (6.22) | 512 (11.13) |
| 23 | Oken E, Radesky JS, Wright RO, et al. Maternal fish intake during pregnancy, blood mercury levels, and child cognition at age 3 years in a US cohort. Am J Epidemiol. 2008;167(10):1171-1181. | 294 (24.50) | 289 (24.08) | 301 (25.08) | 457 (38.08) |
| 24 | Grandjean P, Weihe P, Jørgensen PJ, Clarkson T, Cernichiari E, Viderø T. Impact of maternal seafood diet on fetal exposure to mercury, selenium, and lead. Arch Environ Health. 1992;47(3):185-195. | 279 (9.96) | 274 (9.79) | 285 (10.18) | 432 (15.43) |
| 25 | Wigle DT, Arbuckle TE, Turner MC, et al. Epidemiologic evidence of relationships between reproductive and child health outcomes and environmental chemical contaminants. J Toxicol Environ Health B Crit Rev. 2008;11(5-6):373-517. | 277 (23.08) | 268 (22.33) | 301 (25.08) | 475 (39.58) |
| 26 | Holmes P, James KA, Levy LS. Is low-level environmental mercury exposure of concern to human health?. Sci Total Environ. 2009;408(2):171-182. | 273 (24.82) | 250 (22.73) | 278 (25.27) | 440 (40.00) |
| 27 | Steuerwald U, Weihe P, Jørgensen PJ, et al. Maternal seafood diet, methylmercury exposure, and neonatal neurologic function. J Pediatr. 2000;136(5):599-605. | 271 (13.55) | 261 (13.05) | 292 (14.60) | 447 (22.35) |
| 28 | Bose-O'Reilly S, McCarty KM, Steckling N, Lettmeier B. Mercury exposure and children's health. Curr Probl Pediatr Adolesc Health Care. 2010;40(8):186-215. | 269 (26.90) | 261 (26.10) | 284 (28.40) | 507 (50.70) |
| 29 | Harada M. Congenital Minamata disease: intrauterine methylmercury poisoning. Teratology. 1978;18(2):285-288. | 265 (6.31) | 259 (6.17) | 272 (6.48) | 453 (10.79) |
| 30 | Daniels JL, Longnecker MP, Rowland AS, Golding J; ALSPAC Study Team. University of Bristol Institute of Child Health. Fish intake during pregnancy and early cognitive development of offspring. Epidemiology. 2004;15(4):394-402. | 250 (14.71) | 246 (14.47) | 263 (15.47) | 401 (23.59) |
| 31 | Hightower JM, Moore D. Mercury levels in high-end consumers of fish. Environ Health Perspect. 2003;111(4):604-608. | 250 (15.63) | 240 (15.00) | 264 (16.50) | 414 (25.88) |
| 32 | Castoldi AF, Coccini T, Ceccatelli S, Manzo L. Neurotoxicity and molecular effects of methylmercury. Brain Res Bull. 2001;55(2):197-203. | 246 (12.95) | 234 (12.32) | 270 (14.21) | 401 (21.11) |
| 33 | Crump KS, Kjellström T, Shipp AM, Silvers A, Stewart A. Influence of prenatal mercury exposure upon scholastic and psychological test performance: benchmark analysis of a New Zealand cohort. Risk Anal. 1998;18(6):701-713. | 235 (10.68) | 227 (10.32) | 266 (12.09) | 385 (17.50) |
| 34 | McKeown-Eyssen GE, Ruedy J, Neims A. Methyl mercury exposure in northern Quebec. II. Neurologic findings in children. Am J Epidemiol. 1983;118(4):470-479. | 233 (6.30) | 229 (6.19) | 233 (6.30) | 65 (1.76) |
| 35 | Marsh DO, Clarkson TW, Cox C, Myers GJ, Amin-Zaki L, Al-Tikriti S. Fetal methylmercury poisoning. Relationship between concentration in single strands of maternal hair and child effects. Arch Neurol. 1987;44(10):1017-1022. | 225 (6.82) | 221 (6.70) | 226 (6.85) | 353 (10.70) |
| 36 | Burbacher, TM, Rodier, PM, Weiss B. Methylmercury developmental neurotoxicity: a comparison of effects in humans and animals. Neurotoxicology and teratology. 1990;2(3), 191-202. | 217 (7.23) | 213 (7.10) | 211 (7.03) | 313 (10.43) |
| 37 | Spyker JM, Sparber SB, Goldberg AM. Subtle consequences of methylmercury exposure: behavioral deviations in offspring of treated mothers. Science. 1972;177(4049), 621-623. | 212 (4.42) | 212 (4.42) | 169 (3.52) | 255 (5.31) |
| 38 | Van Oostdam J, Donaldson SG, Feeley M, et al. Human health implications of environmental contaminants in Arctic Canada: A review. Sci Total Environ. 2005;351-352:165-246. | 202 (13.47) | 194 (12.93) | 213 (14.20) | 363 (24.20) |
| 39 | Costa LG, Aschner M, Vitalone A, Syversen T, Soldin OP. Developmental neuropathology of environmental agents. Annu Rev Pharmacol Toxicol. 2004;44:87-110. | 201 (12.56) | 194 (12.13) | 229 (14.31) | 328 (20.50) |
| 40 | Grandjean P, Budtz-Jørgensen E, White RF, et al. Methylmercury exposure biomarkers as indicators of neurotoxicity in children aged 7 years. Am J Epidemiol. 1999;150(3):301-305. | 200 (9.52) | 196 (9.33) | 222 (10.57) | 361 (17.19) |
| 41 | Davidson PW, Myers GJ, Weiss B. Mercury exposure and child development outcomes. Pediatrics. 2004;113(4 Suppl):1023-1029. | 195 (12.19) | 183 (11.44) | 219 (13.69) | 361 (22.56) |
| 42 | Muckle G, Ayotte P, Dewailly E E, Jacobson SW, Jacobson JL. Prenatal exposure of the northern Québec Inuit infants to environmental contaminants. Environ Health Perspect. 2001;109(12):1291-1299. | 194 (10.21) | 192 (10.11) | 197 (10.37) | 292 (15.37) |
| 43 | Davidson, PW, Myers, GJ, Cox, C., et al. Longitudinal neurodevelopmental study of Seychellois children following in utero exposure to methylmercury from maternal fish ingestion: outcomes at 19 and 29 months. Neurotoxicology. 1995;16 (4), 677-688. | 193 (7.72) | 192 (7.68) | 203 (8.12) | 281 (11.24) |
| 44 | Choi BH. The effects of methylmercury on the developing brain. Prog Neurobiol. 1989;32(6):447-470. | 187 (6.03) | 184 (5.94) | 169 (5.45) | 284 (9.16) |
| 45 | Onishchenko N, Karpova N, Sabri F, Castrén E, Ceccatelli S. Long-lasting depression-like behavior and epigenetic changes of BDNF gene expression induced by perinatal exposure to methylmercury. J Neurochem. 2008;106(3):1378-1387. | 185 (15.42) | 173 (14.42) | 192 (16.00) | 246 (20.50) |
| 46 | Mendola P, Selevan SG, Gutter S, Rice D. Environmental factors associated with a spectrum of neurodevelopmental deficits. Ment Retard Dev Disabil Res Rev. 2002;8(3):188-197. | 183 (10.17) | 171 (9.50) | 209 (11.61) | 351 (19.50) |
| 47 | Axelrad DA, Bellinger DC, Ryan LM et al. Dose–response relationship of prenatal mercury exposure and IQ: an integrative analysis of epidemiologic data. Environmental health perspectives. 2007; 115(4), 609-615. | 183 (14.08) | 180 (13.85) | 197 (15.15) | 311 (23.92) |
| 48 | Cernichiari E, Brewer R, Myers GJ et al. Monitoramento de metilmercúrio durante a gravidez: o cabelo materno prediz a exposição do cérebro do feto. Neurotoxicology. 1995; 16 (4), 705-710. | 182 (7.28) | 177 (7.08) | 192 (7.68) | 277 (11.08) |
| 49 | Aschner M, Yao CP, Allen JW, Tan KH. Methylmercury alters glutamate transport in astrocytes. Neurochem Int. 2000;37(2-3):199-206. | 174 (8.70) | 169 (8.45) | 187 (9.35) | 256 (12.80) |
| 50 | Debes F, Budtz-Jørgensen E, Weihe P, White RF, Grandjean P. Impact of prenatal methylmercury exposure on neurobehavioral function at age 14 years. Neurotoxicol Teratol. 2006;28(3):363-375. | 173 (12.36) | 169 (12.07) | 217 (15.50) | 534 (38.14) |
| 51 | Cox C, Clarkson TW, Marsh DO, et al. Dose-response analysis of infants prenatally exposed to methyl mercury: an application of a single compartment model to single-strand hair analysis. Environmental research. 1989; 49(2), 318-332. | 169 (5.45) | 166 (5.35) | 164 (5.29) | 278 (8.97) |
| 52 | Budtz-Jørgensen E, Grandjean P, Weihe P. Separation of risks and benefits of seafood intake. Environ Health Perspect. 2007;115(3):323-327. | 166 (12.77) | 161 (12.38) | 181 (13.92) | 249 (19.15) |
| 53 | Stewart PW, Reihman J, Lonky EI, Darvill TJ, Pagano J. Cognitive development in preschool children prenatally exposed to PCBs and MeHg. Neurotoxicol Teratol. 2003;25(1):11-22. | 165 (6.60) | 159 (6.36) | 174 (6.96) | 263 (10.52) |
| 54 | Cernichiari E, Toribara TY, Liang L, et al. The biological monitoring of mercury in the Seychelles study. Neurotoxicology. 1995;16(4):613-628. | 165 (9.71) | 162 (9.53) | 160 (9.41) | 227 (13.35) |
| 55 | Grandjean P, Satoh H, Murata K, Eto K. Adverse effects of methylmercury: environmental health research implications. Environ Health Perspect. 2010;118(8):1137-1145. | 165 (16.50) | 161 (16.10) | 181 (18.10) | 289 (28.90) |
| 56 | Donaldson SG, Van Oostdam J, Tikhonov C, et al. Environmental contaminants and human health in the Canadian Arctic [published correction appears in Sci Total Environ. 2012 Aug 1;431:437-8). Sci Total Environ. 2010;408(22):5165-5234. | 163 (16.30) | 160 (16.00) | 166 (16.60) | 259 (25.90) |
| 57 | Mahaffey KR, Sunderland EM, Chan HM, et al. Balancing the benefits of n-3 polyunsaturated fatty acids and the risks of methylmercury exposure from fish consumption. Nutr Rev. 2011;69(9):493-508. | 163 (18.11) | 161 (17.89) | 168 (18.67) | 249 (27.67) |
| 58 | Stern AH, Smith AE. An assessment of the cord blood:maternal blood methylmercury ratio: implications for risk assessment. Environ Health Perspect. 2003;111(12):1465-1470. | 162 (9.53) | 158 (9.29) | 175 (10.29) | 264 (15.53) |
| 59 | Lederman SA, Jones RL, Caldwell KL, et al. Relation between cord blood mercury levels and early child development in a World Trade Center cohort. Environ Health Perspect. 2008;116(8):1085-1091. | 162 (13.50) | 159 (13.25) | 171 (14.25) | 224 (18.67) |
| 60 | Strain JJ, Davidson PW, Bonham MP, et al. Associations of maternal long-chain polyunsaturated fatty acids, methyl mercury, and infant development in the Seychelles Child Development Nutrition Study [published correction appears in Neurotoxicology. 2011 Dec;32(6):990]. Neurotoxicology. 2008;29(5):776-782. | 161 (13.42) | 160 (13.33) | 165 (13.75) | 222 (18.50) |
| 61 | Darvill, T, Lonky E, Reihman J, et al. Prenatal exposure to PCBs and infant performance on the fagan test of infant intelligence. Neurotoxicology. 2000;21(6), 1029-1038. | 157 (3.57) | 153 (3.48) | 169 (3.84) | 220 (5.00) |
| 62 | Koos BJ, Longo LD. Mercury toxicity in the pregnant woman, fetus, and newborn infant. A review. Am J Obstet Gynecol. 1976;126(3):390-409. | 157 (7.85) | 157 (7.85) | 144 (7.20) | 261 (13.05) |
| 63 | Magos L, Clarkson TW. Overview of the clinical toxicity of mercury. Ann Clin Biochem. 2006;43(Pt 4):257-268. | 155 (11.07) | 149 (10.64) | 166 (11.86) | 251 (17.93) |
| 64 | Debes F, Budtz-Jørgensen E, Weihe P, White RF, Grandjean P. Impact of prenatal methylmercury exposure on neurobehavioral function at age 14 years. Neurotoxicol Teratol. 2006;28(5):536-547. | 154 (11.00) | 152 (10.86) | 136 (9.71) | 534 (38.14) |
| 65 | Vahter M, Akesson A, Lind B, Björs U, Schütz A, Berglund M. Longitudinal study of methylmercury and inorganic mercury in blood and urine of pregnant and lactating women, as well as in umbilical cord blood. Environ Res. 2000;84(2):186-194. | 151 (7.55) | 148 (7.40) | 170 (8.50) | 290 (14.50) |
| 66 | Stringari J, Nunes AK, Franco JL, et al. Prenatal methylmercury exposure hampers glutathione antioxidant system ontogenesis and causes long-lasting oxidative stress in the mouse brain. Toxicol Appl Pharmacol. 2008;227(1):147-154. | 149 (12.42) | 146 (12.17) | 157 (13.08) | 206 (17.17) |
| 67 | Björnberg, K. A., Vahter, M., Petersson-Grawe, K., et al. Methyl mercury and inorganic mercury in Swedish pregnant women and in cord blood: influence of fish consumption. Environmental Health Perspectives. 2003;111(4), 637-641. | 148 (8.71) | 144 (8.47) | 162 (9.53) | 237 (13.94) |
| 68 | Ha E, Basu N, Bose-O'Reilly S, et al. Current progress on understanding the impact of mercury on human health. Environ Res. 2017;152:419-433. | 147 (49.00) | 142 (47.33) | 161 (53.67) | 236 (78.67) |
| 69 | Spyker JM. Assessing the impact of low-level chemicals on development: behavioral and latent effects. Fed Proc. 1975;34(9):1835-1844. | 145 (3.22) | 142 (3.16) | 117 (2.60) | 188 (4.18) |
| 70 | Davidson PW, Strain JJ, Myers GJ, et al. Neurodevelopmental effects of maternal nutritional status and exposure to methylmercury from eating fish during pregnancy [published correction appears in Neurotoxicology. 2011 Dec;32(6):989]. Neurotoxicology. 2008;29(5):767-775. | 143 (3.25) | 141 (3.20) | 147 (3.34) | 191 (4.34) |
| 71 | Sørensen N, Murata K, Budtz-Jørgensen E, Weihe P, Grandjean P. Prenatal methylmercury exposure as a cardiovascular risk factor at seven years of age. Epidemiology. 1999;10(4):370-375. | 143 (6.81) | 143 (6.81) | 161 (7.67) | 284 (13.52) |
| 72 | Amin-Zaki L, Elhassani S, Majeed MA, et al. Perinatal methylmercury poisoning in Iraq. Am J Dis Child. 1976;130(10):1070-1076. | 143 (11.92) | 143 (11.92) | 146 (12.17) | 222 (18.50) |
| 73 | Marsh DO, Myers GJ, Clarkson TW, Amin-Zaki L, Tikriti S, Majeed MA. Fetal methylmercury poisoning: clinical and toxicological data on 29 cases. Ann Neurol. 1980;7(4):348-353. | 142 (3.55) | 142 (3.55) | 130 (3.25) | 208 (5.20) |
| 74 | Freire C, Ramos R, Lopez-Espinosa MJ, et al. Hair mercury levels, fish consumption, and cognitive development in preschool children from Granada, Spain. Environ Res. 2010;110(1):96-104. | 141 (14.10) | 136 (13.60) | 150 (15.00) | 213 (21.30) |
| 75 | Murata K, Weihe P, Budtz-Jørgensen E, Jørgensen PJ, Grandjean P. Delayed brainstem auditory evoked potential latencies in 14-year-old children exposed to methylmercury [published correction appears in J Pediatr. 2006 Oct;149(4):583-4]. J Pediatr. 2004;144(2):177-183. | 140 (8.75) | 137 (8.56) | 177 (11.06) | 309 (19.31) |
| 76 | Butler Walker J, Houseman J, Seddon L, et al. Maternal and umbilical cord blood levels of mercury, lead, cadmium, and essential trace elements in Arctic Canada. Environ Res. 2006;100(3):295-318. | 140 (10.00) | 137 (9.79) | 146 (10.43) | 228 (16.29) |
| 77 | Clifton JC. Mercury exposure and public health. Pediatr Clin North Am. 2007;54(2):237-viii. | 137 (10.54) | 129 (9.92) | 120 (9.23) | 275 (21.15) |
| 78 | Fernandes Azevedo B, Barros Furieri L, Peçanha FM, et al. Toxic effects of mercury on the cardiovascular and central nervous systems. J Biomed Biotechnol. 2012;2012:949048. | 137 (17.13) | 135 (16.88) | 128 (16.00) | 246 (30.75) |
| 79 | Davis LE, Kornfeld M, Mooney HS, et al. Methylmercury poisoning: long-term clinical, radiological, toxicological, and pathological studies of an affected family. Ann Neurol. 1994;35(6):680-688. | 136 (5.23) | 134 (5.15) | 146 (5.62) | 213 (8.19) |
| 80 | Kajiwara Y, Yasutake A, Adachi T, Hirayama K. Methylmercury transport across the placenta via neutral amino acid carrier. Arch Toxicol. 1996;70(5):310-314. | 134 (5.58) | 130 (5.42) | 145 (6.04) | 206 (8.58) |
| 81 | Vrijheid M, Casas M, Gascon M, Valvi D, Nieuwenhuijsen M. Environmental pollutants and child health-A review of recent concerns. Int J Hyg Environ Health. 2016;219(4-5):331-342. | 131 (32.75) | 127 (31.75) | 140 (35.00) | 205 (51.25) |
| 82 | Grandjean, P., Murata, K., Budtz-Jørgensen, et al. Cardiac autonomic activity in methylmercury neurotoxicity: 14-year follow-up of a Faroese birth cohort. The Journal of pediatrics 2004;144(2), 169-176. | 130 (8.13) | 125 (7.81) | 143 (8.94) | 259 (16.19) |
| 83 | Sheehan MC, Burke TA, Navas-Acien A, Breysse PN, McGready J, Fox MA. Global methylmercury exposure from seafood consumption and risk of developmental neurotoxicity: a systematic review. Bull World Health Organ. 2014;92(4):254-269F. | 129 (21.50) | 126 (21.00) | 142 (23.67) | 195 (32.50) |
| 84 | Johansson C, Castoldi AF, Onishchenko N, Manzo L, Vahter M, Ceccatelli S. Neurobehavioural and molecular changes induced by methylmercury exposure during development. Neurotox Res. 2007;11(3-4):241-260. | 127 (9.77) | 125 (9.62) | 128 (9.85) | 183 (14.08) |
| 85 | Cordier S, Garel M, Mandereau L, et al. Neurodevelopmental investigations among methylmercury-exposed children in French Guiana. Environ Res. 2002;89(1):1-11. | 125 (6.94) | 121 (6.72) | 136 (7.56) | 200 (11.11) |
| 86 | Spyker JM, Smithberg M. Effects of methylmercury on prenatal development in mice. Teratology. 1972;5(2):181-190. | 124 (2.58) | 124 (2.58) | 112 (2.33) | 160 (3.33) |
| 87 | Gundacker C, Fröhlich S, Graf-Rohrmeister K, et al. Perinatal lead and mercury exposure in Austria. Sci Total Environ. 2010;408(23):5744-5749. | 124 (3.02) | 120 (2.93) | 125 (3.05) | 188 (4.59) |
| 88 | Budtz-Jørgensen E, Keiding N, Grandjean P, Weihe P. Confounder selection in environmental epidemiology: assessment of health effects of prenatal mercury exposure. Ann Epidemiol. 2007;17(1):27-35. | 124 (9.54) | 124 (9.54) | 125 (9.62) | 175 (13.46) |
| 89 | Amin-Zaki L, Majeed MA, Elhassani SB, Clarkson TW, Greenwood MR, Doherty RA. Prenatal methylmercury poisoning. Clinical observations over five years. Am J Dis Child. 1979;133(2):172-177. | 124 (12.40) | 123 (12.30) | 117 (11.70) | 206 (20.60) |
| 90 | Li P, Feng X, Qiu G. Methylmercury exposure and health effects from rice and fish consumption: a review. Int J Environ Res Public Health. 2010;7(6):2666-2691. | 123 (5.86) | 110 (5.24) | 126 (6.00) | 165 (7.86) |
| 91 | Rhainds M, Levallois P, Dewailly E, Ayotte P. Lead, mercury, and organochlorine compound levels in cord blood in Québec, Canada. Arch Environ Health. 1999;54(1):40-47. | 123 (12.30) | 121 (12.10) | 126 (12.60) | 176 (17.60) |
| 92 | Buelke-Sam J, Kimmel CA, Adams J, et al. Collaborative Behavioral Teratology Study: results. Neurobehav Toxicol Teratol. 1985;7(6):591-624. | 122 (3.49) | 121 (3.46) | 118 (3.37) | 190 (5.43) |
| 93 | Ostrea EM, Morales V, Ngoumgna E, et al. Prevalence of fetal exposure to environmental toxins as determined by meconium analysis. Neurotoxicology. 2002;23(3):329-339. | 121 (6.72) | 118 (6.56) | 129 (7.17) | 167 (9.28) |
| 94 | Onishchenko N, Tamm C, Vahter M, et al. Developmental exposure to methylmercury alters learning and induces depression-like behavior in male mice. Toxicol Sci. 2007;97(2):428-437. | 120 (4.80) | 117 (4.68) | 121 (4.84) | 166 (6.64) |
| 95 | Myers GJ, Marsh DO, Davidson PW, et al. Main neurodevelopmental study of Seychellois children following in utero exposure to methylmercury from a maternal fish diet: outcome at six months. Neurotoxicology. 1995;16(4), 653-664. | 120 (6.67) | 116 (6.44) | 120 (6.67) | 175 (9.72) |
| 96 | Ask K, Akesson A, Berglund M, Vahter M. Inorganic mercury and methylmercury in placentas of Swedish women. Environ Health Perspect. 2002;110(5):523-526. | 120 (9.23) | 117 (9.00) | 129 (9.92) | 228 (17.54) |
| 97 | Rice DC, Schoeny R, Mahaffey K. Methods and rationale for derivation of a reference dose for methylmercury by the U.S. EPA. Risk Anal. 2003;23(1):107-115. | 119 (7.00) | 116 (6.82) | 127 (7.47) | 198 (11.65) |
| 98 | Coyle I, Wayner MJ, Singer G. Behavioral teratogenesis: a critical evaluation. Pharmacol Biochem Behav. 1976;4(2):191-200. | 115 (2.61) | 114 (2.59) | 90 (2.05) | 166 (3.77) |
| 99 | Maurice-Bourgoin L, Quiroga I, Chincheros J, et al. Mercury distribution in waters and fishes of the upper Madeira rivers and mercury exposure in riparian Amazonian populations. Science of the Total Environment, 2000;260(1-3), 73-86. | 112 (5.60) | 103 (5.15) | 119 (5.95) | 232 (11.60) |
| 100 | Boucher O, Jacobson SW, Plusquellec P, et al. Prenatal methylmercury, postnatal lead exposure, and evidence of attention deficit/hyperactivity disorder among Inuit children in Arctic Québec. Environ Health Perspect. 2012;120(10):1456-1461. | 110 (13.75) | 109 (13.63) | 117 (14.63) | 183 (22.88) |

WoS: WoS:Web of Science; CC: Core Collection

^a^Mean based on the ratio of the numbers of citations and the period since the year of publication up to December 2020
